# Associations of mental disorders and neurotropic parasitic diseases: a meta-analysis in developing and emerging countries

**DOI:** 10.1186/s12889-019-7933-4

**Published:** 2019-12-05

**Authors:** Labanté Outcha Daré, Pierre-Emile Bruand, Daniel Gérard, Benoît Marin, Valerie Lameyre, Farid Boumédiène, Pierre-Marie Preux

**Affiliations:** 1 INSERM, CHU Limoges, UMR_S 1094, Tropical Neuroepidemiology, University of Limoges, Institute of Neuroepidemiology and Tropical Neurology, CNRS FR 3503 GEIST, F-87000 Limoges, France; 2 Access to Medicines, Sanofi, SAG / CSVB, 82 AV Raspail, 94250 Gentilly, France

**Keywords:** Meta-analysis, Association, Co-morbidities, Mental disorders, Neurotropic parasitic diseases

## Abstract

**Background:**

Although they are declining worldwide, neurotropic parasitic diseases are still common in developing and emerging countries. The aim of this study was to estimate the pooled prevalence and pooled association measures of comorbidities between mental disorders (anxiety, depression, bipolar disorder, and schizophrenia) and neurotropic parasitic diseases (malaria, cysticercosis, toxoplasmosis, human African trypanosomiasis, Chagas disease, and human toxocariasis) in developing and emerging countries.

**Methods:**

As the first meta-analysis on this topic, this study was performed in accordance with PRISMA guidelines. The protocol was registered in PROSPERO (N°CRD42017056521). The Medline, Embase, Lilacs, and Institute of Epidemiology and Tropical Neurology databases were used to search for articles without any restriction in language or date. We evaluated the quality of studies independently by two investigators using the Downs and Black assessment grid and pooled estimates using the random-effects method from CMA (Comprehensive Meta Analysis) Version 3.0.

**Results:**

In total, 18 studies published between 1997 and 2016 met our inclusion criteria. We found that the prevalence of anxiety and depression in people suffering from Chagas disease and/or neurocysticercosis was 44.9% (95% CI, 34.4–55.9). In 16 pooled studies that included 1782 people with mental disorders and 1776 controls, toxoplasmosis and/or toxocariasis were associated with increased risk of schizophrenia and/or bipolar disorders (odds ratio = 2.3; 95% CI, 1.7–3.2). Finally, toxocariasis and/or toxoplasmosis were associated with an increased risk of the onset of schizophrenia (odds ratio = 2.4; 95% CI, 1.7–3.4).

**Conclusion:**

Our pooled estimates show that the associations between diseases studied are relatively high in developing and emerging countries. This meta-analysis supports the hypothesis that toxoplasmosis could be the cause of schizophrenia. These findings could prove useful to researchers who want to further explore and understand the associations studied.

## Background

Although parasitic diseases are declining, they are still common in developing and emerging countries. Among the public health issues typically faced by developing and emerging countries (all non high income countries according to World Bank rankings) [1], neurotropic parasitic diseases are very common [2]. Neurotropic parasitic diseases such as malaria, cysticercosis, toxoplasmosis, human African trypanosomiasis (HAT), Chagas disease, and human toxocariasis have a predilection for infesting the central nervous system, which can lead to neurological disorders. Similarly, mental disorders are frequent with pooled 12-month period prevalence estimates of 17.6% (15.5–20.0%) in low and middle-income countries [3]. However, there are few studies regarding the association of mental disorders with neurological parasitoses in these countries.

To date, epidemiological studies have identified several risk factors for the different disease groups studied [4–9]. The table below (Table 1) provides a summary of the main risk factors of interest diseases in this meta-analysis. We have focused our attention on parasitic diseases due to the ever-present burden of these diseases in developing and emerging countries, and we selected the diseases according to their overall burden [5, 11–13]. It is estimated that mental disorders rank third among the most frequent diseases encountered in the world, just after cancer and cardiovascular diseases [14].

**Table 1:** Risk factors of interest diseases

Disease	Risk factor
*Mental disorders*	• Family history • Stressful living conditions • Existence of a chronic disease • Traumatisms • Drug use • Child abuse or neglect and/or lack of social support [4, 5]
*Neurotropic parasitic diseases*	
Human African Trypanosomiasis (HAT) Chagas disease	• Family history of HAT, living near a wetland [6] • Economic, cultural, and human behaviour [7]
Cysticercosis Toxoplasmosis Human Toxocariasis	• Age, home, consumption of undercooked meat, and unwashed fruit or raw vegetables [8] • Bare hand contact with the ground or injury to animals, consumption of poorly washed vegetables [10]

In developing and emerging countries, surveys have suggested that more than 25% of individuals in their lifetime develop one or more mental or behavioural disorders [15]. In the general population, it has been estimated that 3% of people are affected by severe depression, 2% by generalized anxiety disorders and 1% by schizophrenia [16]. Parasitic diseases remain a major burden to developing and emerging countries, although this type of disease has declined globally. In 2015, out of 95 countries and territories in the world where malaria transmission remains high, it is estimated that the number of malaria cases was 214 million (95% CI, 149–303) and that malaria is responsible for 438,000 deaths per year (95% CI, 236000–635,000) mainly in Africa (88%) [17]. In 2012, eight million individuals were already infected with Trypanosoma cruzi, the parasite that causes Chagas disease, in endemic areas of 21 Latin American countries. In addition, the chronic infection caused by this parasite is incurable, can be disabling, and causes more than 10,000 deaths per year [18]. The seroprevalence of toxocariasis varies from 2.4 to 30.0% in Europe [19], but in tropical countries, higher prevalences have been reported: 7.5 to 92.8% in Africa [20, 21], 6.4 to 52.0% in South America [22, 23], and 5.0 to 84.6% in Asia [24, 25]. Human African trypanosomiasis (HAT) affects 60 million inhabitants mainly living in rural areas of 36 endemic sub-Saharan countries in East, West and Central Africa [26]. Toxoplasmosis remains frequent in these countries and even in developed countries [10]. Finally, the agent responsible for cysticercosis, *Taenia solium*, is found mainly in Latin America, Asia, sub-Saharan Africa, and the Indian Ocean region. According to the World Health Organization (WHO), cysticercosis is responsible for 50,000 deaths per year with 2.5 to 5 million adult worm carriers and 50 million cysticercal larvae carriers [27, 28]. Neurocysticercosis, a type of brain damage resulting from cysticercosis, is thought to be a factor responsible for more than 50% of late onset epileptic seizures in developing countries [27].

In recent years, there has been an increasing number of studies on co-morbidities between mental disorders and parasitic diseases, particularly those with neurological tropism such as toxoplasmosis [9], human toxocariasis [29, 30], and cysticercosis [31–34]. However, these have produced heterogenous data and to date, no meta-analysis has been published on the association of mental disorders and parasitic diseases in developing and emerging countries; hence the reason why we decided to perform this study.

## Purpose of the study

The purpose of this study was to estimate the pooled prevalence and pooled association measures of comorbidities between mental disorders and neurotropic parasitic diseases in developing and emerging countries.

## Methods

To perform this meta-analysis, the diseases of interest are neutropic parasitic diseases: malaria, cysticercosis, toxoplasmosis, HAT, Chagas disease, and human toxocariasis [9, 13]. These are forms of parasitosis that have a predilection for infesting the central nervous system and which can result in neurological disorders. Mental disorders of interest were the same as those investigated in the previous meta-analysis [35]. These disorders interfere with thinking, feeling, mood, communication and daily functioning, which typically lead to a reduction in the ability to perform common daily activities, such as caring for family or working [4]. These were: anxiety, depression, bipolar disorder and schizophrenia [5, 11].

Since this meta-analysis is the continuation of a previously published study on the association of mental disorders and chronic physical diseases in developing and emerging countries, its shares the same research strategy, inclusion criteria and selection of articles, data extraction, article quality assessment and statistical analysis as our previous meta-analysis [35].

The protocol for this meta-analysis was recorded in PROSPERO (N° CRD42017056521) and accessible via the following link: http://www.crd.york.ac.uk/PROSPERO. This meta-analysis follows the recommended methodology for the meta-analysis of observational studies [36] and was performed in accordance with Preferred Reporting Items for Systematic Review and Meta-Analysis (PRISMA) guidelines [37].

The search for articles was conducted through four databases: Medline, Embase, Lilacs, and IENT (database of the Institute of Epidemiology and Tropical Neurology of the University of Limoges in France: http://www-ient.unilim.fr/) by LOD, the principal investigator, from February to May 2017 without linguistic or date restrictions.

The same research equation built on Medline below and used for the previous meta-analysis [35] was used in the other databases to search for articles in each of the 139 countries studied:

“***(****“Depressive Disorder”[Mesh] OR “Depression” [Mesh] OR “Anxiety Disorders”[Mesh] OR “Anxiety” [Mesh] OR “Bipolar Disorder”[Mesh] OR “Schizophrenia”[Mesh]****) AND (****“Diabetes metillus”[Mesh] OR “Obesity”[Mesh] OR “Neoplasms”[Mesh] OR “Cardiovascular Diseases”[Mesh] OR “Pulmonary Disease, Chronic Obstructive”[Mesh]* ***OR*** *“Malaria”[Mesh] OR “Cysticercosis”[Mesh] OR “Toxoplasmosis”[Mesh] OR “Toxocariasis”[Mesh] OR “Trypanosomiasis”[Mesh] OR “Chagas Disease”[Mesh]****) AND (****“Name of a country”[Mesh]****)***.” [35]

When searching for articles in the IENT database, which is specifically dedicated to work on neurotrophic parasitosis in the countries of interest of this study, only the free text terms “comorbidity” or “mental health comorbidity” were used. Finally, the registration and selection of articles was done through the Zotéro software.

Every article included in this meta-analysis had to meet the same criteria as the previous meta-analysis, which were: “ be an original article whose full text was available; be a cross-sectional or analytical study; have been conducted on adult patients, both males and females, and on all age groups (age≥15 years); be a study involving either only hospitalised subjects or only non-hospitalised subjects, but not both hospitalised and non-hospitalised subjects at the same time; specify the method of disease diagnosis. For cross-sectional studies, it had to give the prevalence, or the data from which it could be calculated, and for analytical studies, it had to give the association measures or the data from which they could be calculated. “ [35].

As in the previous meta-analysis [35], the revised Downs and Black evaluation grid was used independently [38, 39] by two researchers (LOD and PEB) to assess the quality of the studies included in this meta-analysis and the data were extracted for each article by LOD, the principal investigator. The statistical tests were performed with a significance threshold of 5% using Comprehensive Meta-Analysis (CMA) Version 3.0 [40]. *Q-test* and *I*^*2*^ [41, 42] were performed to assess the heterogeneity of the studies included in our pooled estimates. The DerSimonian-Laird random effects technique [43] was then used to calculate the pooled estimates and the results obtained were presented in forest plot. In order to investigate publication bias, we constructed a funnel plot and performed a Duval and Tweedie adjustment and filling test [43], and, Egger regression [44]. The robustness of our results was tested using the sensitivity test, which consisted of subtracting the study with the highest weight among the studies included in a pooled estimate and subtracting the lowest quality studies among the studies from a pooled estimate. Finally, we conducted subgroup analyses for the variables: original disease, associated disease, type of subject and continent.

## Results

### General results

**Fig. 1:**
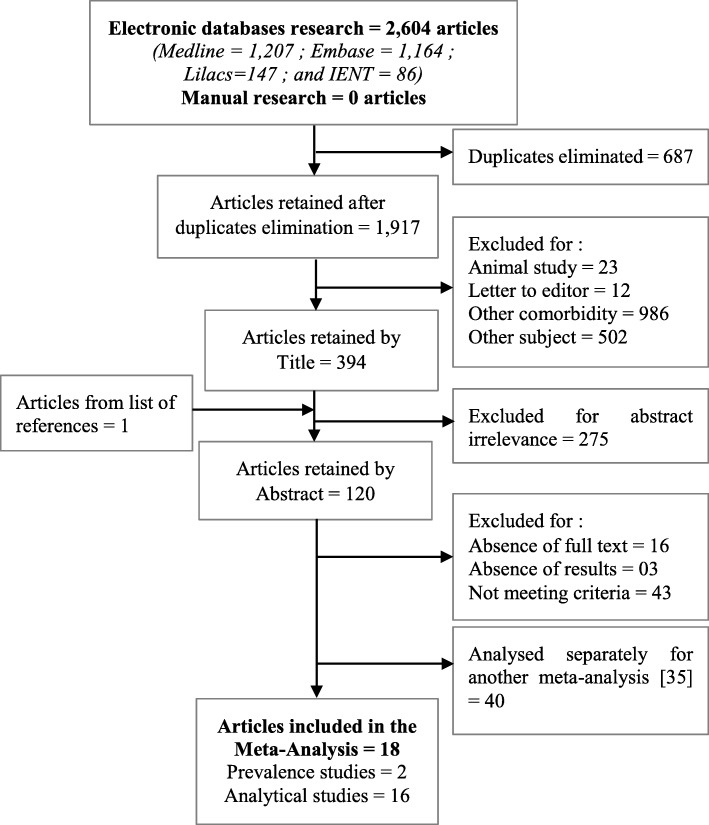
Research strategy flow chart for the meta-analysis of associations of mental disorders and neurotropic parasitic diseases in developing and emerging countries

Out of the 2604 different articles in English, French, Spanish, Portuguese, Chinese, and Russian, initially identified for co-morbidities between mental health and chronic physical diseases, and for co-morbidities between mental health and neurotropic parasitic diseases, 18 articles were included in our meta-analysis (Fig. 1). Among these 18 articles of co-morbidity studies on mental disorders and neurotropic parasitic diseases [45–62] published between 1997 and 2016 and meeting our inclusion criteria, two were prevalence studies in individuals with neurotropic parasitic diseases who were screened for mental disorders, and sixteen were analytical studies in individuals with mental disorders who were screened for neurotropic parasitic diseases (Table 2 and Table 3).

**Table 2:** Characteristics of prevalence studies of associations of mental disorders and neurotropic parasitic diseases

Reference	Continent	Subjects type	Original disease	Associated disease	Positive (n)	Total (N)	Diagnostic method	F/M	Average age
Ozaki et al. [61] Qual Life Res, 2011	Asia	Non-hospitalised	Chagas disease	Anxiety and Depression	45	110	BDI	56/54	51 ± 13
Forlenza et al. [52] J Neurol Neurosurg Psychiatry, 1997	America	Non-hospitalised	Neurocysticercosis	Depression	20	38	PSE; MMSE; SADS-L; MSE	20/18	36.7

Description of data:

*BDI* Beck Depression Inventory, *PSE* Present State Examination, *MMSE* Mini Mental State Examination, *SADS-L* Schedule for Affective Disorders and Schizophrenia-Lifetime, *MSE* Mental Status Examination, *F* Female, *M* Male, Age in years

**Table 3:** Characteristics of analytical studies of associations of mental disorders and neurotropic parasitic diseases

Reference	Continent	Subjects type	Original disease	Associated disease	Study Design	Positive (n)	Total (N)	OR	Diagnostic method	F/M	Age (case / control)
Daryani et al. [49] Trop Biomed, 2010	Asia	Non-hospitalised	Schizophrenia	*T. gondii* infection	Case - Control	28 25	80 99	–	ELISA *“*IgG & IgM*” (Germany)* ELISA reader (*USA)*	–	32.95 ± 10.05 33.76 ± 10.50
Alipour et al. [45] Iran J Parasitol, 2011	Asia	Hospitalised	Schizophrenia	*T. gondii* infection	Case - Control	42 23	62 62	–	IFA and ELISA “IgG” (Denmark) ELISA reader (BioTek, USA)	59/65	37.54 ± 9.75 37.24 ± 10.24
Karabulut et al. [56] J Chin Med Assoc, 2015	Asia	Hospitalised	Schizophrenia	*T. gondii* infection	Case - Control	–	85 60	1008	ELISA Kits (Spain) Triturus system (Spain)	60/85	41.73 ± 12.07 40.45 ± 9.49
Khademvatan et al. [58] Jundishapur J Microbiol, 2014	Asia	Non-hospitalised	Schizophrenia	*T. gondii* infection	Case - Control	34 53	100 200	–	ELISA “IgG (USA)	139/161	36.39 ± 10.28 25.04
Hamidinejat et al. [53] Int J Infect Dis, 2010	Asia	Hospitalised	Schizophrenia	*T. gondii* infection	Case - Control	–	98 94	2,99	ELISA kits “IgG and M” (USA)	–	33 (Case & Control)
Tamer et al. [62] Adv Ther, 2008	Asia	Hospitalised	Schizophrenia	*T. gondii* infection	Case - Control	16 5	40 37	–	ELISA (Germany)	–	Cas: 33 Control: –
Alvarado-Esquivel et al. [47] Parasitol Int, 2011	America	Hospitalised	Schizophrenia	*T. gondii* infection	Case - Control	–	50 150	4,44	EIA kit “Ig G & M” (USA)	–	45.12 ± 11.5 45.1 ± 11.4
Cetinkaya et al. [48] Schizophr Bull, 2007	Asia	Non-hospitalised	Schizophrenia	*T. gondii* infection	Case - Control	66 33	100 100	–	ELISA kit “Ig G” (France)	103/97	37.25 ± 11.51 37.3 ± 5.78
Omar et al. [60] Korean J Parasitol, 2015	Asia	Non-hospitalised	Schizophrenia	*T. gondii* infection	Case - Control	52 10	101 55	–	ELISA PCR (Qpcr) (Germany)	74/82	41.1 ± 10.9 45.3 ± 14.5
Esshili et al. [51] Psychiatry Res, 2016	Africa	Non-hospitalised	Schizophrenia	*T. gondii* infection	Case - Control	–	246 117	2,54	ELISA kit “IgG” (France) ELISA reader	89/274	40.5 ± 10.2 38.6 ± 10.4
Juanah et al. [54] Am J Infect Dis, 2013	Asia	Hospitalised	Schizophrenia	*T. gondii* infection	Case - Control	–	88 88	2,01	EIA “IgG & M” (Italy) Microtiter plate reader (DYNEX, MRX)	66/110	39.42 (Case & Control)
Kheirandish et al. [59] Arch Clin Infect Dis, 2016	Asia	Hospitalised	Schizophrenia and bipolar disorders	Toxoplasmosis	Case - Control	49 and 54 65 and 65	85 170	–	EIA “IgG” Microplate reader (USA)	85/150	37.2 (Case & Control)
Emelia et al. [50] Trop Biomed, 2012	Asia	Hospitalised	Schizophrenia	Toxocariasis	Case - Control	54 49	144 144	–	ELISA “Ig G” (USA) ELISA reader (SKANIT Software)	–	–
Alvarado-Esquivel et al. [46] Int Int J Biomed Sci, 2014	America	Hospitalised	Schizophrenia	Toxocariasis	Case - Control	1 3	50 100	–	EIA “IgG” (U.S.A.)	–	45.12 ± 11.5 45.5 ± 13.1
Kaplan et al. [55] Yonsei Yonsei Med J, 2008	Asia	Hospitalised	Schizophrenia	Toxocariasis	Case - Control	45 2	98 100	–	ELISA kit “IgG & M” (Germany) ELISA microtiter plate reader	95/103	38 ± 11 35 ± 8
Khademvatan et al. [57] J Med Sci Faisalabad, 2013	Asia	Hospitalised	Bipolar disorders (I)	Toxoplasma	Case - Control	–	117 200	0,78	ELISA “IgG & M” (USA)	162/155	33.93 ± 11.87 33.88 ± 11.45

Description of data:

Kheirandish et al., [59]: Cases = 49 patients with schizophrenia and 54 patients with bipolar disorders, Controls = 85 for schizophrenia cases and 85 for bipolar disorder cases

*T. gondii* Toxoplasma gondii, *GHQ* General Health Questionnaire, *ELISA* Immunosorbant Enzyme-Linked Assay, *IFA* Immunofluorescent Assay, *EIA* Enzyme Immuno Assay Enzyme Immuno Assay, *PCR* Polymerase Chain Reaction, *qPCR* quantitave Polymerase Chain Reaction, *F* Female, *M* Male, Age in years

In the prevalence studies examined in this meta-analysis, the female to male sex ratio was 1.1 and the mean age was 43.9 ± 7.5 years. In the analytical case-control studies, the female to male sex ratio was 0.7, while the mean age was 36.0 ± 11.0 years for cases, and 37.6 ± 10.5 years for controls. In the 18 analytical studies conducted, 11 were hospitalised patients. Non-hospitalised patients were studied in two prevalence studies and in the 7 remaining analytical studies. With the Downs and Black assessment grid, the mean quality score for the prevalence studies was found to be 18.5 ± 2 (maximum score of 22), while the mean for the case-control studies was found to be 16.4 ± 3.1 (maximum score of 25) (Additional file 1: Table S1 and Additional file 2: Table S2).

### Main findings

**Fig. 2:**
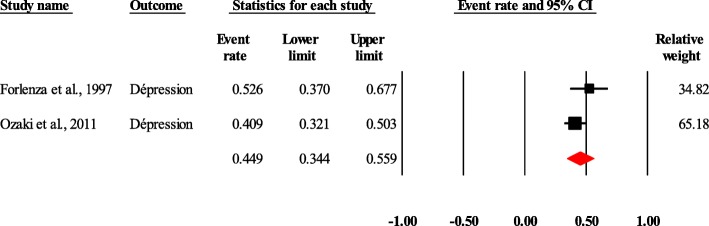
Forest plot of the pooled prevalence of anxiety and/or depression in people with Chagas disease and/or neurocysticercosis. Heterogeneity: Q = 1.56, df = 1, *p* = 0.21, I^2^ = 36.03

**Fig. 3:**
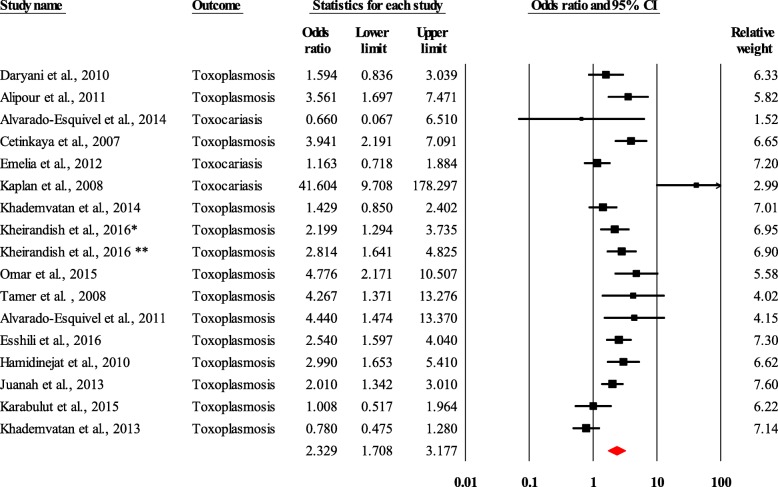
Forest plot of the pooled odds ratio of toxoplasmosis and/or toxocariasis in people with schizophrenia and/or bipolar disorders, Heterogeneity: Q = 62.67, df = 16, *p* < 0.0001, I^2^ = 74.47, Kheirandish et al., 2016*: Schizophrenia, Kheirandish et al., 2016**: Bipolar disorders

In the prevalence studies, the diseases found were anxiety and/or depression for mental disorders, and Chagas disease and neurocysticercosis for neurotropic parasitic diseases. There were 16,610 subjects enrolled in these studies. The prevalence of depression in the 148 individuals with Chagas disease and neurocysticercosis was 44.9% (95% CI, 34.4–55.9) (Fig. 2). With regard to neuotropic parasitic diseases, toxoplasmosis and toxocariasis were found. In the 16 pooled studies that included 1782 people with schizophrenia and/or bipolar disorders, and 1776 controls. Mental disorders (schizophrenia and/or bipolar disorders) were associated with an increased risk of toxoplasmosis and/or toxocariasis (odds ratio = 2.3; 95% CI, 1.7–3.2) (Fig. 3).

**Fig. 4:**
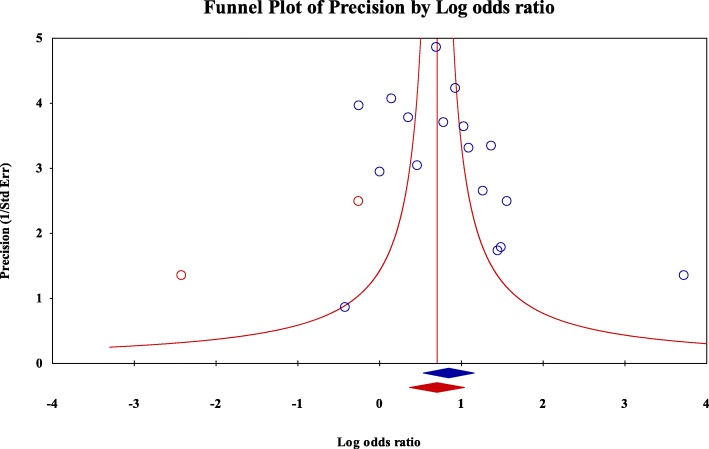
Funnel plot showing found and missing analytical studies of associations of mental disorders and neurotropic parasitic diseases

The distribution of the pooled studies was illustrated using a funnel plot. Based on Duval and Tweedie’s “trim and fill” visual method, we found that 2 analytical studies were missing (Fig. 4). Afterwards, we found that the intercept result obtained by the Egger test was 2.4 (95% CI, − 0.5 - 5.3 / *p* = 0.09), which confirms the existence of publication bias.

From the included analytical studies, we found that the pooled odds ratio of the association of schizophrenia and/or bipolar disorders and toxoplasmosis and/or toxocariasis in cases (people with mental disorders) versus controls after sensivity analyses did not changes. It varied from 2.3 (95% CI, 1.7–3.2) to 2.3 (95% CI, 1.7–3.3) when the study with the greatest weight, Esshili et al. [51], was withdrawn. And it became 2.2 (95% CI, 1.6–3.1) when Cetinkaya et al. [48] and Hamidinejat et al. [53], the studies with the lowest quality scores, were withdrawn.

**Fig. 5:**
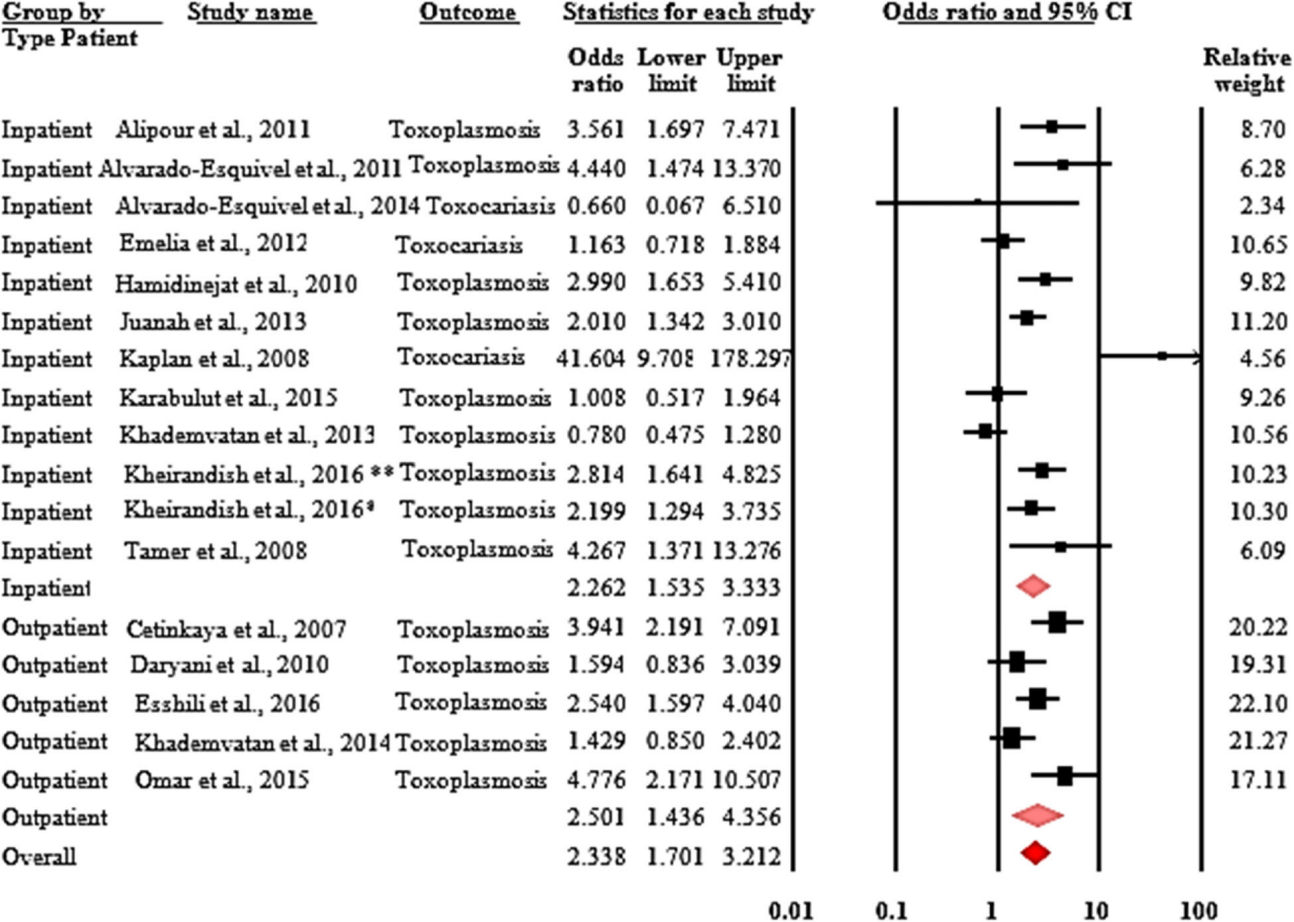
Forest plot of the pooled odds ratio of associations of mental disorders and neurotropic parasitic diseases by type of subjects (hospitalised and non-hospitalised). Heterogeneity: Q = 62.67, df = 16, *p* < 0.0001, I2 = 74.47, Kheirandish et al., 2016*: Schizophrenia, Kheirandish et al., 2016**: Bipolar disorders

Analysis of the selected subgroups by type of disease investigated, type of associated disease, type of subjects included, and continent allowed us to show the following findings: the odds ratio of the association between schizophrenia and toxocariasis was 2.7 (95% CI, 1.1–7.0) in people with schizophrenia compared to the control group. And the association between mental disorders (schizophrenia and/or bipolar disorders) with toxoplasmosis was 2.3 (95% CI, 1.7–3.2) in people with schizophrenia and/or bipolar disorders compared to the control group. For the association between schizophrenia and neurotropic parasitic diseases (toxocariasis and/or toxoplasmosis) the odd ratio was 2.4 (95% CI, 1.7–3.4). Co-morbidities between mental disorders (schizophrenia and/or bipolar disorders) and neurotropic parasitic diseases (toxoplasmosis and/or toxocariasis) had closely similar odds ratios in both hospitalised and non-hospitalised people. The odds ratio was 2.3 (95% CI, 1.5–3.3) for the association between mental disorders (schizophrenia and/or bipolar disorders) and neurotropic parasitic diseases (toxoplasmosis and/or toxocariasis) when comparing cases with schizophrenia and/or bipolar disorders and controls. It was 2.5 (95% CI, 1.4–4.4) for the association between mental disorders (schizophrenia and/or bipolar disorders) and toxoplasmosis when comparing cases with schizophrenia and/or bipolar disorders and controls (Fig. 5). Finally, the subgroup analysis by continent showed that in Asia, the odds ratio of co-morbidities between mental disorders (schizophrenia and/or bipolar disorders) and neurotropic parasitic diseases (toxoplasmosis and/or toxocariasis) was 2.3 (95% CI, 1.6–3.3).

## Discussion

We focused on developing and emerging countries studied due to their high burden in the selected diseases. For mental disorders, these were anxiety, depression, bipolar disorder, and schizophrenia [5, 11] and for parasitic diseases with neurological tropism, these were malaria, cysticercosis, toxoplasmosis, HAT, Chagas disease, and human toxocariasis [9, 13].

In general, the diagnosis of diseases in the studies selected in this meta-analysis was performed using acceptable diagnostic techniques. Indeed, the quality of diagnostic techniques used to screen for mental disorders has been developed in accordance with DSM-5 and ICD-10 [63]. Nevertheless, it has been shown, as in the study of Kirkil et al., that the questionnaire used can also influence the results in terms of diagnosis [64]. The variety of questionnaires used in the studies included in this meta-analysis can be explained by the need to adapt the questionnaire to the population studied [65–73], as is usually recommended. Regarding laboratory diagnostic techniques, it is recommended to use two complementary diagnostic tests (one very sensitive and therefore very specific) and to have the samples handed by two different technicians who do not know the clinical condition of the subject [74]. However, most of the studies included in this meta-analysis generally use only one fairly sensitive and specific diagnostic method at a time, which is not the best practice. This can be partly explained by financial constraints related to the cost of diagnostic tests, which are often quite expensive in low-income countries, and by the nature of the sample size, which increases the burden of work. This may be acceptable for this type of disease, since parasitic diseases are very commonly found in these countries.

The quality scores of the studies included in this meta-analysis are in line with those found by other authors [75]. There was publication bias and great heterogeneity in our estimates. In both the prevalence studies and the analytical studies, the quality scores were all largely above the average score (i.e. above half the maximum score: 11 for prevalence studies and 12.5 for case-control studies). But since our estimates did not change after we performed the sensitivity analysis. In our pooled estimates after withdrawing the study with the highest weight on the one hand, and the study with the lowest quality score on the other, using the two recommended methods, the sensitivity test had almost no impact on our results and the results remained robust. However, the different levels of heterogeneity found in the pooled estimates after analysis of the selected subgroups (type of original disease, type of associated disease, type of subjects included, and continent) implied that there were other covariates in play and that the latter could be the source of these heterogeneities.

This meta-analysis revealed that, despite the small number of studies included, the prevalence of anxiety and/or depression was almost 50% in people with Chagas disease and/or neurocysticercosis. “In addition, toxocariasis and/or toxoplasmosis was associated with an increased risk of schizophrenia and/or bipolar disorders (odds ratio = 2.3). More specifically, through subgroup analysis, we were able to show that toxocariasis (odds ratio = 2.7) and toxocariasis and/or toxoplasmosis (odds ratio = 2.4) were associated with increased risk of schizophrenia. In hospitalised subjects, the results of the subgroup analysis showed that in the presence toxocariasis and/or toxoplasmosis, the increased risk of mental disorders (schizophrenia and/or bipolar disorders) was 130%, and, in non-hospitalised subjects the increased risk of mental disorders (schizophrenia and/or bipolar disorders) in presence of toxoplasmosis was 150%. This similarity of results in the two types of subjects might reflect the difficulties of access to mental and neurological health care in developing and emerging countries, with mental and neurological diseases being grossly under-diagnosed, and suffering both from a low ranking in terms of public health priority.

Due to the well-known neurotropic characteristics of *Toxoplasma gondii*, it is commonly accepted that many psychiatric symptoms, like mental retardation, may be due to *Toxoplasma gondii* infections [76]. Much interest exists in determining a causative relationship between this parasite and some mental disorders, in particular schizophrenia [77] and bipolar disorders [78]. Our investigation into the presence of IgG antibodies in people with mental disorders produced results that are similar to those obtained by other authors. People living with schizophrenia were found to have a risk ratio of 1.43 to 2.73 for toxoplasmosis and/or toxocariasis [77, 79], while people with bipolar disorders were found to have a risk ratio of 1.26 to 1.52 [78, 79].

The analytical studies on the associations of mental disorders with neurotropic parasitic diseases didn’t allow some statistical analyses, such as subgroup analysis. Only Asia, which accounted for most of the studies, made it possible to estimate that, in presence of toxoplasmosis and/or toxocariasis, there was a 130% increased risk of the onset of schizophrenia and/or bipolar disorders. The lack of data on mental disorders in developing and emerging countries for other continents could potentially be explained by the lack of medical consultations for people with mental disorders, and the lack of knowledge and skills regarding mental illness among primary healthcare professionals; it could also be due to the insufficient number of appropriate health centres, which, when they exist, do not seem to be accessible or have staff trained to manage mental disorders and neurotropic parasitic diseases. Religion and traditional beliefs among communities can also be additional barriers to the diagnosis of mental disorders in these countries, with these diseases often getting attributed to spiritual causes. It has been reported that up to 80% of people with mental disorders and their families might prefer to seek care from religious leaders, traditional healers or exorcist-priests [80]. Finally, poverty and the lack of national and international funding for mental health often aggravate the situation. As a result, a high proportion of people with severe mental disorders (76 to 85%) do not receive treatment in developing and emerging countries [81].

Our results suggest that further studies on the co-morbidities of mental disorders and neurotropic parasitic diseases in developing and emerging countries may be needed to highlight the true estimations of these often-neglected diseases, especially in these countries.

Despite our efforts to limit bias, our study has some limitations that were already described in our previous meta-analysis [35] which shared the same method.

The limitations of this work are related to the small sizes of the different study samples included in our pooled estimates. The fact that the identification of mental disorders was not always confirmed by a specialist in each of the studies is also a limitation. Asia, which accounted for many of the studies, could also well be an obstacle in the generalization of our results to other continents. Finally, the lack of studies meeting all inclusion criteria could be considered as a limitation in the calculation of some estimates for the co-morbidities studied.

Nevertheless, these criteria have enabled us to provide reliable and generalizable pooled estimates for these co-morbidities. This meta-analysis was actually carried out according to the PRISMA 2015 guidelines and the recommendation for conducting sensitivity analyses by subtracting the study with the highest weight and the studies with the lowest quality among the studies included in each pooled estimate.

## Conclusion

In this meta-analysis, the results show that the pooled estimates of co-morbidities between mental disorders and neurotropic parasitic diseases in developing and emerging countries are relatively high. Most of the included studies were conducted in Asia. In conclusion, our findings support the hypothesis that toxoplasmosis could be the cause of schizophrenia and/or bipolar disorders. We hope that this meta-analysis, the first of its kind to focus on developing and emerging countries, can provide some guidance to researchers to further explore and understand the associations between mental disorders and neurotropic parasitic diesases in developing and emerging countries.

## Supplementary information

**Additional file 1 **: **Table S1.** Characteristics of quality scores of prevalence studies. Global quality (Items: 1–2–3-5-6-7-9-10); External validity (Items:11–12-13); Results bias (Items: 15–16–18-20); Confusion and selection bias (Item: 25); Power (Item: 27) and S: Quality score.

**Additional file 2 **: **Table S2**. Characteristics of quality scores of analytical studies. Global quality (Items: 1–2–3-5-6-7-9-10); External validity (Items:11–12-13); Results bias (Items: 15–16–18-20); Confusion and selection bias (Item: 25); Power (Item: 27) and S: Quality score.

## Data Availability

All information generated or analyzed during this meta-analysis is available in the additional data provided by the first author.

## Abbreviations

BDI: Beck Depression Inventory
CMA: Comprehensive Meta Analysis
DSM-5: Diagnostic and Statistical Manual of Mental Disorders
EIA: Enzyme Immuno Assay Enzyme Immuno Assay
ELISA: Immunosorbant Enzyme-Linked Assay
GHQ: General Health Questionnaire
HAT: Human African Trypanosomiasis
ICD-10: International Classification of Diseases
IENT: Institute of Epidemiology and Tropical Neurology
IFA: Immunofluorescent Assay
MMSE: Mini Mental State Examination
MSE: Mental Status Examination
PCR: Polymerase Chain Reaction
PRISMA: Preferred Reporting Items for Systematic Review and Meta-Analysis
PSE: Present State Examination
qPCR: quantitave Polymerase Chain Reaction
SADS-L: Schedule for Affective Disorders and Schizophrenia-Lifetime
