# Supplementary material for: Associations of mental disorders and neurotropic parasitic diseases: a meta-analysis in developing and emerging countries
Source: BMC Public Health. 2019 Dec 5;19:1645. doi: 10.1186/s12889-019-7933-4 (PMC6896488; doi:10.1186/s12889-019-7933-4)
Supplement: Supplementary file 1 — Additional file 1 : Table S1. Characteristics of quality scores of prevalence studies. Global quality (Items: 1–2–3-5-6-7-9-10); External validity (Items:11–12-13); Results bias (Items: 15–16–18-20); Confusion and selection bias (Item: 25); Power (Item: 27) and S: Quality score. [file 12889_2019_7933_MOESM1_ESM.docx]

| Reference | Item  *1 2 3 5 6 7 9 10 11 12 13 15 16 18 20 25 27* | | | | | | | | | | | | | | | | | S |
| --- | --- | --- | --- | --- | --- | --- | --- | --- | --- | --- | --- | --- | --- | --- | --- | --- | --- | --- |
| Forlenza et al. [[55](#_ENREF_55)] | 1 | 1 | 1 | 2 | 1 | 1 | 1 | 1 | 1 | 1 | 0 | 0 | 1 | 1 | 1 | 1 | 5 | 21 |
| Ozaki et al. [[64](#_ENREF_64)] | 1 | 1 | 1 | 2 | 1 | 1 | 1 | 1 | 0 | 1 | 1 | 0 | 1 | 1 | 1 | 1 | 1 | 16 |
