# Supplementary material for: Associations of mental disorders and neurotropic parasitic diseases: a meta-analysis in developing and emerging countries
Source: BMC Public Health. 2019 Dec 5;19:1645. doi: 10.1186/s12889-019-7933-4 (PMC6896488; doi:10.1186/s12889-019-7933-4)
Supplement: Supplementary file 2 — Additional file 2 : Table S2. Characteristics of quality scores of analytical studies. Global quality (Items: 1–2–3-5-6-7-9-10); External validity (Items:11–12-13); Results bias (Items: 15–16–18-20); Confusion and selection bias (Item: 25); Power (Item: 27) and S: Quality score. [file 12889_2019_7933_MOESM2_ESM.docx]

| Reference | Item  *1 2 3 5 6 7 9 10 11 12 13 15 16 18 20 21 22 25 26 27* | | | | | | | | | | | | | | | | | | | | S |
| --- | --- | --- | --- | --- | --- | --- | --- | --- | --- | --- | --- | --- | --- | --- | --- | --- | --- | --- | --- | --- | --- |
| Daryani et al. [[52](#_ENREF_52)] | 1 | 1 | 1 | 2 | 0 | 0 | 1 | 0 | 0 | 1 | 1 | 0 | 1 | 1 | 1 | 1 | 1 | 0 | 1 | 3 | 17 |
| Alipour et al. [[48](#_ENREF_48)] | 1 | 1 | 1 | 1 | 1 | 1 | 1 | 1 | 0 | 1 | 0 | 0 | 1 | 1 | 1 | 1 | 1 | 0 | 1 | 2 | 17 |
| Alvarado-Esquivel et al. [[49](#_ENREF_49)] | 1 | 1 | 1 | 2 | 1 | 0 | 1 | 1 | 1 | 1 | 1 | 0 | 1 | 1 | 1 | 1 | 1 | 0 | 1 | 4 | 21 |
| Alvarado-Esquivel et al. [[50](#_ENREF_50)] | 1 | 1 | 1 | 1 | 1 | 1 | 1 | 1 | 0 | 1 | 1 | 0 | 1 | 1 | 1 | 1 | 1 | 0 | 1 | 4 | 20 |
| Cetinkaya et al. [[51](#_ENREF_51)] | 1 | 1 | 1 | 2 | 1 | 0 | 1 | 0 | 0 | 1 | 0 | 0 | 1 | 0 | 1 | 0 | 0 | 0 | 1 | 1 | 12 |
| Emelia et al. [[53](#_ENREF_53)] | 1 | 1 | 1 | 0 | 1 | 1 | 1 | 1 | 1 | 1 | 0 | 0 | 1 | 1 | 1 | 1 | 1 | 0 | 1 | 1 | 16 |
| Esshili et al. [[54](#_ENREF_54)] | 1 | 1 | 1 | 1 | 1 | 1 | 1 | 1 | 0 | 1 | 0 | 0 | 1 | 1 | 1 | 1 | 0 | 0 | 1 | 0 | 14 |
| Hamidinejat et al. [[56](#_ENREF_56)] | 1 | 1 | 1 | 0 | 1 | 1 | 1 | 0 | 0 | 0 | 0 | 0 | 1 | 1 | 1 | 0 | 0 | 0 | 1 | 1 | 11 |
| Juanah et al. [[57](#_ENREF_57)] | 1 | 1 | 2 | 1 | 1 | 1 | 1 | 1 | 0 | 1 | 1 | 0 | 1 | 1 | 1 | 1 | 0 | 1 | 1 | 1 | 18 |
| Kaplan et al. [[58](#_ENREF_58)] | 1 | 1 | 1 | 1 | 1 | 1 | 1 | 1 | 0 | 1 | 1 | 0 | 1 | 1 | 1 | 1 | 0 | 0 | 1 | 2 | 17 |
| Karabulut et al. [[59](#_ENREF_59)] | 1 | 1 | 1 | 0 | 1 | 1 | 1 | 1 | 0 | 1 | 0 | 0 | 1 | 1 | 1 | 1 | 0 | 0 | 1 | 0 | 13 |
| Khademvatan et al. [[61](#_ENREF_61)] | 1 | 1 | 1 | 2 | 1 | 1 | 1 | 1 | 0 | 1 | 0 | 0 | 1 | 1 | 1 | 1 | 1 | 1 | 1 | 2 | 19 |
| Khademvatan et al. [[60](#_ENREF_60)] | 1 | 1 | 1 | 0 | 1 | 1 | 1 | 1 | 0 | 1 | 0 | 0 | 1 | 1 | 1 | 1 | 1 | 0 | 1 | 3 | 17 |
| Kheirandish et al. [[62](#_ENREF_62)] | 1 | 1 | 1 | 2 | 1 | 1 | 1 | 1 | 1 | 1 | 1 | 0 | 1 | 1 | 1 | 1 | 1 | 1 | 1 | 3 | 22 |
| Omar et al. [[63](#_ENREF_63)] | 1 | 1 | 1 | 0 | 1 | 1 | 1 | 1 | 1 | 1 | 0 | 0 | 1 | 1 | 1 | 1 | 0 | 0 | 1 | 0 | 14 |
| Tamer et al. [[65](#_ENREF_65)] | 1 | 1 | 1 | 2 | 1 | 0 | 1 | 0 | 0 | 1 | 0 | 0 | 1 | 1 | 1 | 1 | 1 | 0 | 1 | 0 | 14 |
